# Kikuchi-Fujimoto Disease Associated with Myasthenia Gravis: A Case Report

**DOI:** 10.1155/2010/903252

**Published:** 2010-08-11

**Authors:** Olukayode Onasanya, David Nochlin, Victor Casas, Leema Reddy Peddareddygari, Raji P. Grewal

**Affiliations:** Laboratory of Neurogenetics, New Jersey Neuroscience Institute, JFK Medical Center, 65 James Street, Edison NJ, 08820, USA

## Abstract

Kikuchi-Fujimoto disease is a self-limited benign condition of unknown etiology characterized by cervical lymphadenopathy, fever, and leucopenia. An autoimmune hypothesis has been suggested and an association with systemic lupus erythematosus, Sjogren's disease, and antiphospholipid syndrome has been noted. We report a 27-year-old male who presented for evaluation of weakness and he was diagnosed with seropositive generalized myasthenia gravis and underwent a thymectomy. He was stable until five months post-thymectomy, when he developed a high fever associated with nontender cervical lymphadenopathy, chills, and night sweats. Histopathology of a cervical lymph gland biopsy was compatible with Kikuchi-Fujimoto lymphadenitis. He improved spontaneously and was asymptomatic at the followup six months later. Our case expands the association of Kikuchi-Fujimoto disease with autoimmune disorders to include myasthenia gravis.

## 1. Introduction

Kikuchi-Fujimoto disease (KFD) also known as histiocytic necrotizing lymphadenitis or necrotizing granulomatous lymphadenitis is a rare disease of unclear etiology. This condition was first described in Japanese patients by Kikuchi and Fujimoto independently but simultaneously in 1972 [1, 2]. Since then, it has been described in diverse populations from North America and Europe [2]. It affects women more often than men with a ratio of 4 : 1 and with age of onset typically 20–30 years. Clinically, it is characterized by fever, cervical lymphadenopathy, leucopenia, and other constitutional symptoms [3]. The incidence is estimated to range between 0.5% and 5% of all cases of pathologically analyzed lymphadenopathy [4]. Pathologically, the disease is characterized by coagulative necrosis, histiocytic infiltrate, loss of nodal architecture, and absence of polymorphonuclear leukocytes [2]. To diagnose KFD histologically, the following criteria are used: (a) patchy, irregular areas of eosinophilic necrosis in the paracortex and/or the cortex (brick red necrosis), (b) pronounced fragments of nuclear dust distributed in an irregular fashion through the area of necrosis, (c) absence of granulocytes and a paucity of plasma cells, (d) clusters of plasmacytoid T cells, and (e) numerous immunoblasts predominantly of T-cell phenotype [2, 5].

The cause of KFD is unclear but various antigen-induced hyperimmune reactions and/or an autoimmune process where apoptosis occurs have been proposed in the pathophysiology [4]. Although no direct cause and effect relationship has been established, several viruses including Epstein-Barr virus (EBV), parvovirus B19, and human herpes virus-six have been implicated as these potential antigens [1]. Dorfman and Berry suggest that KFD may be an attenuated form of systemic lupus erythematosus (SLE) as the similarities in the lymph node histology are striking [1]. Kikuchi-Fujimoto disease has been associated with a number of autoimmune diseases such as SLE, mixed connective tissue disease, antiphospholipid antibody syndrome, thyroiditis, polymyositis, scleroderma, autoimmune hepatitis, and Still's disease. In this paper, we expand the association of KFD with another autoimmune disease, myasthenia gravis (MG).

## 2. Case Report

A 27-year-old man presented to the Neuromuscular clinic at the New Jersey Neuroscience Institute/JFK Medical Center for evaluation of weakness. After a neurological examination and the further investigations were performed, he was diagnosed with seropositive generalized MG. He underwent a thymectomy, and pathological examination showed mild follicular hyperplasia of thymic tissue. Post-thymectomy, he was treated with 2.0 grams/kg intravenous immunoglobulin (IVIG) every month in addition to pyridostigmine. He was neurologically stable, and about approximately five months later he developed a persistent high-grade fever (101.4–104.0 F) associated with chills and rigor. 

The fever was higher in the evening, associated with night sweats, and it persisted even after the IVIG was discontinued. There was no associated skin rash, weight loss, cough, chest pain, or shortness of breath. His physical examination was unremarkable except for bilaterally palpable nontender anterior and posterior cervical, supraclavicular, and axillary lymphadenopathy. There was no epitrochlear or inguinal lymph nodal involvement and no hepatosplenomegaly. 

Routine investigations showed the following abnormalities: white blood cell count (WBC): 1.71 (4.5–11 × 10^9^/liter), serum lactate dehydrogenase (LDH): 832 (100–225 U/l), alanine transaminase and aspartate aminotransferase were 85 (8–30) U/L and 132 (0–55) U/L, respectively; erythrocyte sedimentation rate (ESR) was 132 (0–15 mm/hr); the C-reactive protein (CRP) was 20.95 (0–5 mg/L).

The following investigations were either normal or negative: blood cultures for bacteria, viruses and fungi, serology against EBV, cytomegalovirus, parvovirus B19, histoplasmosis, borrelia burgdorferi, human immunodeficiency virus, hepatitis B virus, hepatitis C virus, treponema pallidum, antinuclear antibody test, anti-double-stranded DNA antibody, anti-sm antibody, and anti-Sjögren's syndrome A (anti-SSA) and anti-Sjögren's syndrome B (anti-SSB) antibodies.

**Figure 1:**
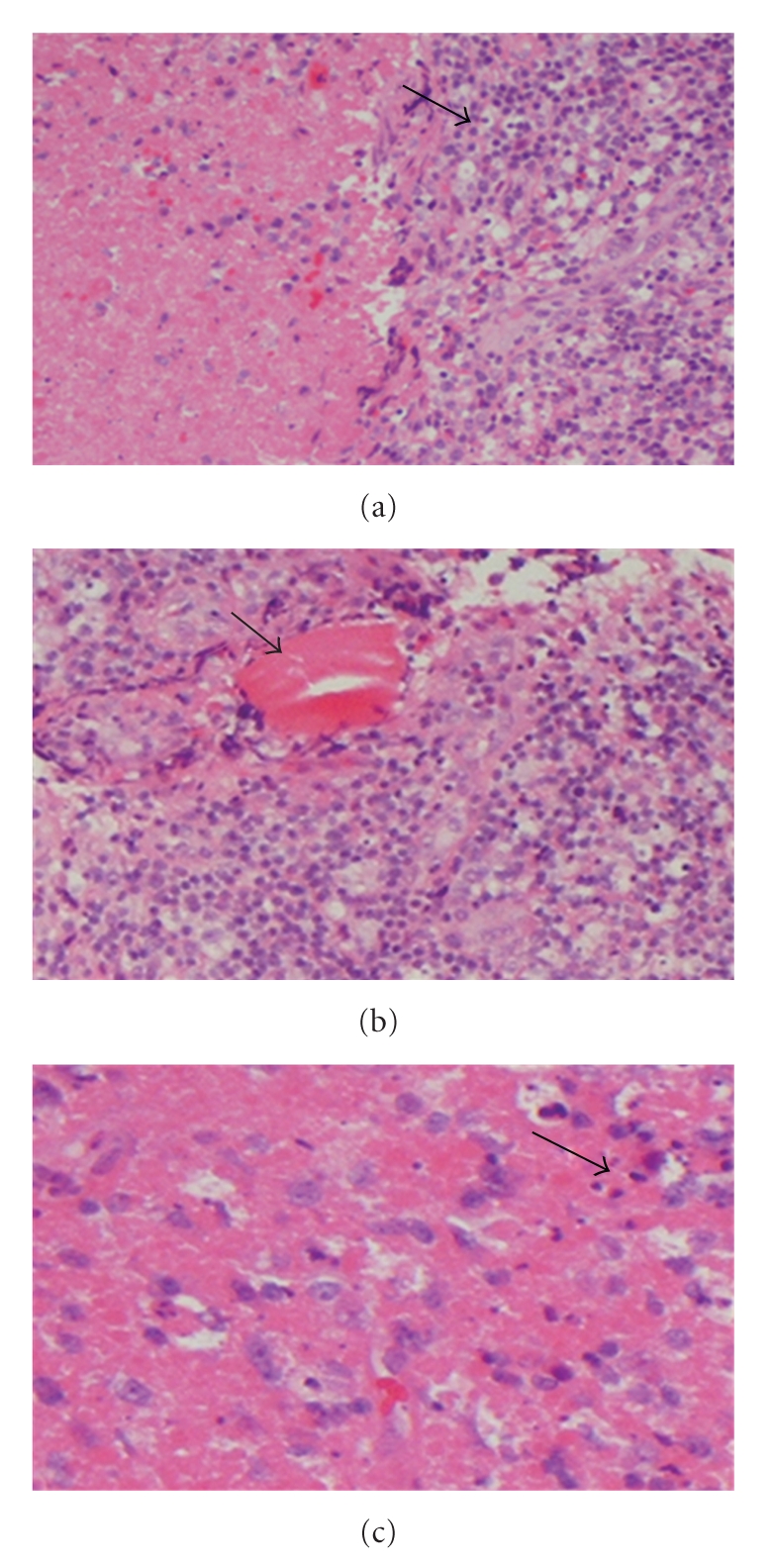
Hematoxylin- and eosin-stained section showing histological appearance of a lymph node biopsy in medium (a) and high (b, c) magnifications. (a) Lymph node showing necrosis and mononuclear cell infiltrate (arrow). (b) Brick red necrosis (arrow). (c) Apoptosis and nuclear dust (arrow). (a, b, and c) x10 (original magnification).

A spiral computed tomography (CT) scan of the neck with contrast confirmed many clusters of almost all groups of cervical lymphadenopathy, the largest measuring 2.29 × 1.24 × 2.71 cm. There was no mediastinal or hilar adenopathy on chest CT. A fine needle aspiration biopsy of a cervical lymph gland was performed, and cultures for bacterial (including acid-fast bacillus) and fungal organisms were negative. However, histological examination showed a necrotizing histiocytic lymphadenitis consistent with Kikuchi-Fujimoto lymphadenitis (Figure 1).

Symptomatic treatment of the fever was instituted and lymphadenopathy resolved spontaneously. Six months after lymph node biopsy, the cervical and axillary lymphadenopathy had resolved. In addition, routine serum chemistries including his WBC count and liver functions tests had all returned to normal.

## 3. Discussion

 Kikuchi-Fujimoto disease is an enigmatic, benign, and self-limited disease presenting with an acute or subacute onset and progression over two to three weeks. Lymphadenopathy is the most frequent sign with cervical lymphadenopathy observed 74%–90% of time, especially in the posterior cervical triangle. The nodes are firm in consistency and may be tender. In 30%–50% of patients, fever is the presenting symptom [4, 6]. Other symptoms and signs include splenomegaly, weight loss, arthralgia, and skin rash. The latter is seen in one third of patients at presentation leading to confusion in the clinical scenario with infectious mononucleosis, SLE, and lymphoma. There is leucopenia in 25%–50% of cases with nonspecific findings of elevated LDH, transaminases, ESR, and CRP [1, 7].

Our patient has both the clinical and histological criteria confirming the diagnosis of KFD. There is a report of possible treatment of KFD with IVIG [8]. However, our patient developed KFD while on IVIG, raising doubts about its therapeutic utility in this condition.

This is the first report of a patient with both KFD and MG and expands the growing list of autoimmune diseases that can be associated with KFD. Although KFD undergoes a spontaneous resolution in 3–6 months, long-term monitoring is recommended as recurrence is known to occur in 3% of cases [4].
